# Delayed Diagnosis and Multi-TKI Intolerance: A Case Report of CML Concurrent With COVID-19

**DOI:** 10.3389/fonc.2022.921587

**Published:** 2022-06-08

**Authors:** Chengxin Luan, Haixia Wang, Junjie Zhou, Xiaoyu Ma, Zhangbiao Long, Xin Cheng, Xiaowen Chen, Ruixiang Xia, Jian Ge

**Affiliations:** Department of Hematology, The First Affiliated Hospital of Anhui Medical University, Hefei, China

**Keywords:** COVID-19, chronic myeloid leukemia, delayed diagnosis, tyrosine kinase inhibitors, case report

## Abstract

**Introduction:**

The hematological manifestations of corona virus disease 2019 (COVID-19) can confound the diagnosis and therapy of other diseases. In this paper, we firstly reported a case of chronic myeloid leukemia (CML) of delayed diagnosis and intolerance to tyrosine kinase inhibitors (TKIs) concurrent with COVID-19.

**Case Presentation:**

A 56-year-old female was diagnosed as COVID-19 with no obvious leukocytosis [white blood cell (WBC), ≤17 × 10^9^/L] or splenomegaly until ablation of the virus. Bone marrow aspiration was conducted to establish the diagnosis of CML. She accepted an adjusted dosage of imatinib initially and had to suspend it after myelosuppression (day 41). After hematopoietic therapy, imatinib was given again (day 62), but she was still non-tolerant, and nilotinib at 150 mg twice a day was prescribed from day 214. At just about 4 weeks later, nilotinib was discontinued due to myelosuppression. Then, it was reduced to 150 mg per day and was re-initiated (day 349), but she was still non-tolerant to it. Similarly, from day 398, flumatinib at 200 mg per day was tried, but she was non-tolerant. Her white blood cell or platelet count fluctuated markedly with poor therapeutic response. Considering that she was relatively tolerant and responsive to imatinib, the medication was re-initiated at 200 mg and reduced to 100 mg per day. Her follow-up revealed stable WBC and PLT counts. The latest BCR-ABL-210/ABL was decreased to 0.68% at about 6 months after imatinib was re-initiated, which means an improved response.

**Conclusion:**

The offset effect between CML and SARS-CoV-2 infection was supposed to be the underlying mechanism for the absence of leukocytosis or splenomegaly. The impact of immune network by SARS-CoV-2 preserved and disrupted the patient’s response to TKIs despite the virus’ ablation. We suggest that a continued elevation of basophils may be a useful indicator for CML concurrent with COVID-19, and individualized treatment with adjusted dosage and suitable type of TKIs should be considered to improve the patient’s health outcome.

## Introduction

Since its first outbreak in Wuhan, China, in 2019, corona virus disease 2019 (COVID-19), caused by severe acute respiratory syndrome coronavirus 2 (SARS-CoV-2), has been sweeping across the globe and causing great losses to international health and economy, with more than 6 million deaths globally as of the beginning of 2022 (1). Though in some regions, such as the mainland of China, the epidemic situation has been limited to sporadic outbreaks, COVID-19 is still a pandemic around the world, with no downward trend especially in the United States, India, and Brazil. Compared with severe acute respiratory syndrome coronavirus in 2003, COVID-19 has a lower mortality rate but a higher basic reproduction number as both symptomatic and asymptomatic transmissions endure (2, 3). It was also reported that the recovered patients could test positive again and recrudesce in areas where lockdowns have already worked (4, 5). Meanwhile, the successive emergence of new variants outpaced the vaccine development and endowed a greater ability to spread (2, 6). These epidemiological characteristics result in the difficulty to its containment and cause the medical community to accept that human beings will coexist with COVID-19, just like influenza virus (3, 7). It is foreseeable that COVID-19 will continuously impact on healthcare and bring into focus on the diagnosis and treatment of conditions concurrent with its infection.

COVID-19 infection commonly involves the respiratory system and causes clinical manifestations such as cough, fever, pulmonary inflammation, hypoxemia, and acute respiratory distress syndrome. However, emerging data indicates that other systems can also be affected, especially in severe cases, due to the fact that the viruses enter host cells *via* ligand receptor-mediated endocytosis and that their corresponding receptor-binding domain is expressed virtually on any organ of the human body, thus precipitating a systemic immunological hyper-response or injury (2, 3, 8). Therefore, understanding the impact of COVID-19 on systems other than the respiratory system is also important. Its hematological manifestations are non-specific, from the more common leukopenia or lymphopenia to the less observed coagulation disorders, thrombocytopenia, and leukocytosis, all of which can complicate the diagnosis and treatment of hematological diseases (3, 8, 9). Here we report a case of chronic myeloid leukemia (CML) of delayed diagnosis and intolerance to various tyrosine kinase inhibitors (TKIs) due to SARS-CoV-2 infection. Then, we discuss the underlying mechanism and propose a strategy in aiming to better diagnose and treat CML concurrent with COVID-19 infection.

## Case Presentation

**Figure 1:**
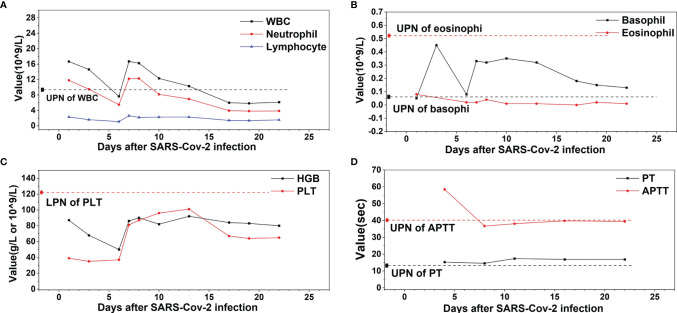
The profile of white blood cell (WBC), hemoglobin (HGB), platelet (PLT), prothrombin time (PT), and activated partial thromboplastin time (APTT) of the patient during the therapy course of COVID-19. **(A)** WBC, neutrophil, and lymphocyte. **(B)** Basophil and eosinophil. **(C)** HGB and PLT. **(D)** PT and APTT. ULN, upper limit of normal, LLN, lower limit of normal.

A 56-year-old female of Han nationality presented to the local hospital in February 2020 (Anhui, China) with fever (38°C), without chills, and coughing without expectoration for 2 days; no clear underlying diseases or inducing factors were found. Upon auscultation, there were sporadic moist crackles in both lungs. She had no relevant medical, family, and psycho-social history. The blood test revealed that the count of white blood cells (WBC) was 16.67 × 10^9^/L, dominated by neutrophils (11.81 × 10^9^/L), and the level of hemoglobin (HGB) was 87 g/L, while the number of platelets (PLT) was 39 × 10^9/^L. C-reactive protein (CRP) was 59.9 mg/L. The coagulation function test revealed the prothrombin time (PT) as 16.3 s, the activated partial thromboplastin time (APTT) as 78.0 s, and fibrinogen (Fib) as 3.43 g/L. A computed tomography (CT) of the chest showed bilateral patchy ground-glass opacities, thickened pleura with calcification on the right side, and a small amount of encapsulated effusion in the right pleural cavity. COVID-19 was suspected, and an oropharyngeal (OP) swab was performed and tested positive for SARS-CoV-2 by real-time quantitative polymerase chain reaction (RT-qPCR). She was transported to the isolation ward and accepted antiviral treatment (lopinavir, ritonavir, and interferon), oxygen inhalation, corticosteroids, and intravenous immunoglobulin. About a week later, her condition worsened with persistent hypoxemia, chest distress, and tachycardia, and her case was rated as severe type (4, 10). The laboratory examination revealed that WBC was 7.63 × 10^9^/L, dominated by neutrophils (5.49 × 10^9/^L), HGB was 50 g/L, PLT was 37 × 10^9^/L, CRP was 28 mg/L, PT was 15.3 s, APTT was 58.5 s, and Fib was 3.4 g/L. The CT indicated an extension of pulmonary inflammation, and she was transferred to a superior hospital after infusion of 1.5 units of packed red blood cells to address her anemia. In the superior hospital, the patient’s clinical condition improved—without hypoxemia, chest distress, tachycardia, and moist crackles—after antiviral treatment and supportive care were provided. At about 2 weeks later, the laboratory examination revealed that the WBC was 6.12 × 109/L, dominated by neutrophils (3.85 × 10^9/^L), HGB was 80 g/L, PLT was 65 × 10^9^/L, CRP was 4.5 mg/L, PT was 16.9 s, APTT was 39.4, and Fib was 2.85 g/L. The blood test and the coagulation function test of the patient during the therapy course of SARS-CoV-2 infection are presented in **Figure 1**. As the CT indicated, there was absorption of pulmonary inflammation, and the repeated OP swabs showed SARS-CoV-2 to be negative; she was discharged to a local hospital for quarantine.

During quarantine, all OP swabs were negative, and the repeated laboratory work showed persistent leukocytosis, anemia, thrombocytopenia, and elevation in basophil count. The physical examination showed no hepatosplenomegaly, no sternal tenderness, and no lymphadenectasis. To figure out the underlying disease, she had undergone bone marrow aspiration in March 2020, BCR-ABL fusion gene detection by RT-qPCR, and chromosomal analysis, which confirmed the presence of BCR-ABL-210 fusion gene and Philadelphia translocation, thus establishing the diagnosis of CML (chronic phase). To control leukocytosis, hydroxyurea at 0.5 g twice a day was prescribed from March 2020, until leukopenia developed in May 2020, and thus the patient was admitted to our department for further treatment.

**Figure 2:**
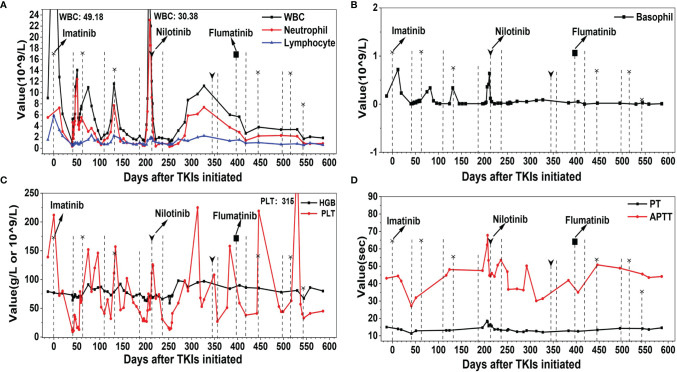
The profile of white blood cell (WBC), hemoglobin (HGB), platelet (PLT), prothrombin time (PT), and activated partial thromboplastin time (APTT) of the patient during the therapy course of tyrosine kinase inhibitors (TKIs). **(A)** WBC, neutrophil, and lymphocyte. **(B)** Basophil. **(C)** HGB and PLT. **(D)** PT and APTT. Dotted line with and without head indicate the initiation and stopping of TKI, respectively. The shape of the heads indicates the type of TKIs.

Bone marrow aspiration was repeated to confirm the diagnosis of CML [chronic phase and intermediate risk by Sokal, Hasford, the European Treatment and Outcome Study (EUTOS) scoring, and the EUTOS long-term survival score systems, BCR-ABL-210/ABL 96.63% (international scale, IS)]. The laboratory examination revealed that WBC was 9.06 × 10^9^/L, dominated by neutrophils (5.55 × 10^9^/L), HGB was 79 g/L, PLT was 139 × 10^9^/L, PT was 15.1 s, APTT was 43.1 s, and Fib was 3.09 g/L at 2 days after admission. The WBC increased to 49.18 × 10^9^/L on May 28, 2020, and an adjusted dosage of imatinib at 300 mg per day was initiated due to concerns over myelosuppression. When PLT decreased to 9 × 10^9^/L on July 8, 2020, imatinib was stopped and recombinant human interleukin-11 was given. When the WBC decreased to 2.52 × 10^9^/L (neutrophils: 1.74 × 10^9^/L) on July 10, 2020, granulocyte colony-stimulating factor (G-CSF) was given. About 3 weeks later, the WBC and the PLT counts recovered, and imatinib at 300 mg per day was given again in combination with interleukin-11. On September 15, 2020, the WBC dropped to 2.4 × 10^9^/L, HGB was 80 g/L, and PLT was 41 × 10^9^/L. Imatinib was suspended and then re-initiated at a reduced dosage of 200 mg per day on October 9, 2020. On December 1, 2020, the WBC plunged to 2.2 × 10^9^/L, HGB was 75 g/L, and PLT was 43 × 10^9^/L. Imatinib was discontinued and BCR-ABL-210/ABL was a 24.89% (IS). The patient’s response was evaluated to be poor, and thus she was recommended to a second-generation TKI. After about 4 weeks of therapy with G-CSF and interleukin-11, an adjusted dosage of nilotinib at 150 mg twice a day was prescribed on December 28, 2020. About 4 weeks later, the WBC further declined to 1.85 × 10^9^/L, HGB was 66 g/L, and PLT was 31 × 10^9^/L, so nilotinib was discontinued for about 4 months without any TKI treatment. On February 2, 2021, BCR-ABL-210/ABL was 137.21% (IS). Reduced nilotinib at 150 mg per day was re-initiated on May 12, 2021 but had to be stopped within about 1 week as her PLT decreased to 43 × 10^9^/L. At the end of June 2021, another second-generation TKI, flumatinib at 200 mg per day, was given—but again she was non-tolerant though flumatinib was reported to have caused lower rates of myelosuppression compared with imatinib (11). She was admitted to our department back and forth and took or discontinued TKIs with G-CSF and/or interleukin-11 intermittently, and her WBC or PLT count fluctuated markedly, with recurrent coagulation dysfunction which was monitored to decrease the risk of bleeding (**Figure 2**). Bone marrow biopsy was repeated and indicatory of active bone marrow hyperplasia (nearly 80%), with obvious signs of myelocytic leukemia and fibrosis. OP swabs were performed several times; all turned out negative.

So far, the patient was non-tolerant to three types of TKIs. The last resort in the TKI arsenal at our department would be dasatinib, which cannot be administered for its impairment of platelet aggregation (12). Considering that she was relatively tolerant and responsive to imatinib, the medication was re-initiated at 200 mg per day from August 17, 2021, and this was reduced to 100 mg per day from November 23, 2021 BCR-ABL-210/ABL was decreased to 54.821% (IS) on November 9, 2021 (84 days after imatinib was re-initiated) and to 0.68% (IS) on February 22, 2022 (189 days after imatinib was re-initiated). Her follow-up examination revealed stable WBC (around 2.5 × 10^9^/L), HGB (around 90 g/L), PLT (around 50 × 10^9^/L), and normal coagulation function. Improved response was indicated, and the patient is satisfied with the current therapeutic regimen and is under follow-up.

## Discussion

COVID-19 could cause systemic immune response and injury to the whole body. The hematopoietic system is liable to a significant impact in patients with a concurrent severe systemic disease (3, 8). SARS-CoV-2 invades host human cells by binding to the angiotensin-converting enzyme 2 receptors, which are primarily expressed in the lungs, heart, and gastrointestinal tract and abundantly existent on the surface of lymphocytes (13). During the incubation period or the early phase of COVID-19, non-specific symptoms or only respiratory symptoms are present. As the infection progresses, a “cytokine storm” may form, other systems are involved, and lymphopenia develops. Though the mechanism of SARS-CoV-2 to lymphopenia has not been fully unveiled, two factors may contribute: viral infection and lysis of lymphocytes. Moreover, “cytokine storm”-related lymphocyte apoptosis and substantial cytokine activation also lead to the atrophy of lymphoid organs, including the spleen (3). Therefore, lymphopenia has been the most common hematological change and regarded as a potential factor for diagnosis and a predictor of COVID-19 prognosis (3, 14). Notably, most of the studies indicated that lymphocytopenia was significantly associated with acute respiratory distress syndrome development, ICU support requirement, and poor prognosis (3). Leukopenia, leukocytosis, and pancytopenia have also been reported (3, 15, 16). Coagulation abnormalities have also been reported in COVID-19 patients with a severe form of the disease, presenting with prolonged PT, prolonged APTT, and decreased platelet count (3, 17, 18). While the underlying mechanism has not been elucidated, immune deregulation, endothelial dysfunction, and pro-inflammatory cytokines may play a similar role as in other systemic coagulopathies, *e.g*., disseminated intravascular coagulation and thrombotic microangiopathy. These coagulation abnormalities have been regarded as deteriorated conditions and indicative of an increased risk of death (3, 14, 19).

**Figure 3:**
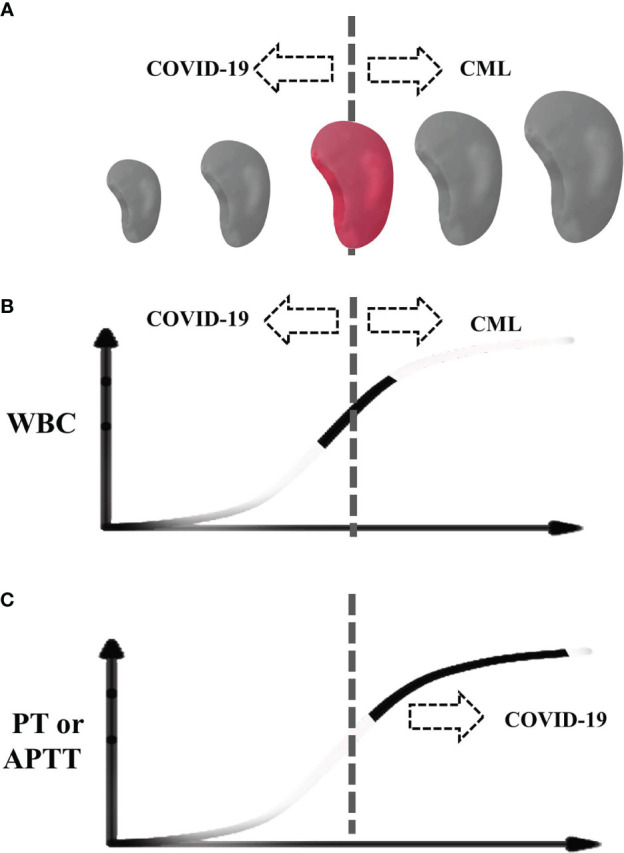
Schematic diagram of our case during COVID-19. **(A)** The spleen size. **(B)** White blood cell. **(C)** Prothrombin time and activated partial thromboplastin time. The dotted line indicates the center of normal size or normal range. Colored spleen and solid curve indicate the range of the patient.

Taken together, the infection of SARS-CoV-2 can affect the diagnosis of hematological diseases. The typical manifestations of CML include fatigue, fever, an enlarged spleen, feeling full, *etc.* The laboratory examinations of CML in the chronic phase mainly reveal leukocytosis dominated by granulocytes, low red blood cell count, and normal platelet count. Abnormal white blood cell count and enlarged spleen are the most important indicators of CML. In our case, the SARS-CoV-2 infection led to leukopenia and atrophy of the spleen; therefore, as illustrated in **Figure 3**, neither obvious leukocytosis nor splenomegaly had emerged due to the offset effect between CML and SARS-CoV-2 infection. Besides this, the coagulation abnormalities and thrombocytopenia in this case further confounded the clues of CML. Only after the ablation of COVID-19 did these classic features of CML emerge and indicated the possibility of CML. However, by pursuing a careful retrospective study of the case, we found that an increased level of basophils was not overridden by COVID-19, which may be a useful indicator for CML concurrent with SARS-CoV-2 infection. To the best of our knowledge, this is the first report on the difficulty of diagnosing CML due to a COVID-19 infection.

Aside from the challenges of diagnosis, COVID-19 can also affect the treatment of hematological diseases and *vice versa*. Cases of SARS-CoV-2 that infected established hematological diseases have been reported; even a case of complete spontaneous remission of lymphoma was reported, and anti-tumor immune response triggered by SARS-CoV-2 was proposed as the underlying mechanism (20, 21). However, most cases indicated that the concurrence of COVID-19 with hematological diseases added difficulty to the therapy or led to poor outcomes due to the immune deficiency caused by the malignancy itself or anticancer treatments or the immune abnormality medicated by COVID-19, which includes CML, and some suggested a possible protective role of the TKIs against the virus (22–29). Specially though, having recovered from COVID-19, the patient in our study remained extremely non-tolerant to four TKIs. We hypothesize that the impact of the viral infection on the immune network may endure, disrupting the patient’s response to TKIs.

## Concluding Remarks

SARS-CoV-2-associated pathological changes of the bone marrow and the blood could mask the features of hematological diseases and result in delayed diagnosis. For the first time, we reported a CML case of delayed diagnosis and intolerance to multi-TKIs due to a co-occurring COVID-19. We suggest that the continued elevation of basophils may be a useful indicator for CML concurrent with SARS-CoV-2 infection, and individualized treatment with an adjusted dosage of TKIs should be considered to improve the patient’s health outcome. However, the conclusion based on a single case and limited literature may be arbitrary and inconclusive. Finally, in view of the rapidly emerging SARS-CoV-2 variants, more attention deserves to be attached to its concurrency with serious diseases, such as hematological malignancies, and further studies of the mechanisms may help delineate novel strategies to meet the diagnostic and therapeutic challenges in this pandemic era.

## Data Availability Statement

The original contributions presented in the study are included in the article. Further inquiries can be directed to the corresponding author.

## Ethics Statement

This study has been approved by the ethics committee of the First Affiliated Hospital of Anhui Medical University. The patient provided her written informed consent to this article.

## Author Contributions

CL and HW were healthcare providers of the patient; they collected and analyzed the data and wrote the paper. JZ, XM, ZL, XC, and XWC were healthcare providers of the patient; they help collect data. RX helped revise the paper. JG is the person in charge of the group and revised the paper. All authors contributed to the article and approved the submitted version.

## Funding

This work was supported by the Natural Science Foundation of Anhui province (grant number 2008085QH365), the Key Research and Development Project of Anhui Province (grant number 201904a07020057), the Research Foundation of Anhui Medical University (grant number 2020xkj166), the Clinical Trial Initiative Projects of The First Affiliated Hospital of Anhui Medical University (grant numberLCYJ2021YB009), and the Research Foundation of Anhui Institute of Translational Medicine (grant number 2021zhyx-C32).

## Conflict of Interest

The authors declare that the research was conducted in the absence of any commercial or financial relationships that could be construed as a potential conflict of interest.

## Publisher’s Note

All claims expressed in this article are solely those of the authors and do not necessarily represent those of their affiliated organizations, or those of the publisher, the editors and the reviewers. Any product that may be evaluated in this article, or claim that may be made by its manufacturer, is not guaranteed or endorsed by the publisher.
